# Intravesical thermochemotherapy in the treatment of high-risk and very high-risk non-muscle-invasive urothelial bladder cancer: a single-arm study

**DOI:** 10.1007/s11255-023-03924-3

**Published:** 2024-02-08

**Authors:** Antonín Brisuda, Jakub Horňák, Barbora Žemličková, Jaromír Háček, Marek Babjuk

**Affiliations:** 2nd Faculty of Medicine, Charles University, V Úvalu 84, 150 06 Prague 5, Czech Republic

**Keywords:** Adjuvant instillations, Intravesical thermochemotherapy, Non-muscle-invasive bladder cancer, Urothelial carcinoma

## Abstract

**Aim:**

Intravesical thermochemotherapy, also known as HIVEC (Hyperthermic Intra-VEsical Chemotherapy), represents an alternative adjuvant topical treatment for non-muscle-invasive urothelial bladder cancer (NMIBC). High-risk (HR) and very HR tumors carry a substantial risk of recurrence and progression. In this study, we present our own results using HIVEC as an alternative to unavailable Bacillus Calmette–Guérin (BCG) vaccine in the treatment of such groups of patients.

**Methods:**

During the period of November 2014–June 2022, a total of 47 patients with HR and very HR NMIBC underwent treatment with HIVEC after transurethral resection. They were given an induction of 6 instillations with/without a maintenance. The aim was to evaluate the time to recurrence, event-free survival (recurrence or progression), as measured by Kaplan–Meier analysis, the effect of maintenance treatment and other factors on survival (log-rank test and multivariable Cox regression analysis), and complications.

**Results:**

The median follow-up for patients who did not experience an event was 32 months. The median time to HR (high grade and/or T1 tumor) recurrence in those who recurred was 15 months. The survival rate without HR recurrence at 12, 24, and 48 months was 84, 70, and 59%, respectively. Progression was detected in 10.6% of patients, which translated to 89% of patients living without progression after 24 months. Maintenance treatment (defined as more than six instillations) and presence of CIS significantly correlated with risk of HR recurrence (Hazard ratio 0.34 and 3.12, respectively). One female patient underwent salvage cystectomy due to contractory bladder, and 19.1% of patients experienced transient lower urinary tract symptoms.

**Conclusion:**

Based on our experience, HIVEC represents an adequate and safe alternative treatment for HR and very HR NMIBC in situations where BCG is not available or radical cystectomy is not an option for the patient. However, high-quality data from prospective randomized studies are still lacking, and thus, thermochemotherapy should still be regarded as an experimental treatment modality.

## Introduction

High-risk (HR) and very HR non-muscle-invasive urothelial bladder cancer (NMIBC) frequently recurs, with a 60% risk in 12 months according to EORTC, and bears a substantial risk of disease progression [1]. According to an individual patient data analysis of 3401 patients treated with TURBT ± intravesical chemotherapy, the risk of disease progression was 11 and 44% in 5 years, respectively [2]. Early radical cystectomy is recommended for very HR disease [3]. The most effective adjuvant treatment for HR tumors remains the intravesical Bacillus Calmette–Guérin (BCG) vaccine with a maintenance protocol, which has been used since the 1970s [4]. Any study investigating alternative adjuvant treatments in this indication should therefore have at least one arm with BCG vaccine [5]. In case of unavailability of BCG, the treatment outcomes should be at least correlated with recent data from large groups of patients treated for HR tumors with BCG [6].

Hyperthermia induces a variety of biological effects on cells, rendering them more susceptible to cross-linking agents such as MMC and cisplatin. One notable impact is the enhancement of blood flow and increased vessel permeability, facilitating a greater delivery of chemotherapeutic drugs to the tumor [7]. Hyperthermia also influences cellular membrane properties, making them more fluid, and upregulates the copper transporter CTR1, which is pivotal for cisplatin transportation into the cell [8]. Additionally, in the context of the bladder wall, hyperthermia is proposed to assist in overcoming its typically impermeable structure, thus aiding in surpassing drug transport barriers for bladder tumors [9]. Once the drugs have entered the cells, hyperthermia employs another mechanism to enhance the efficacy of cisplatin and MMC, specifically by inhibiting DNA repair processes [10]. In an immunohistological study, a reduction in proliferation activity (Ki-67 antibody activity) and p53 activity highlighted the potential role of HIVEC as a promising intravesical treatment [11].

There are no specific contraindications for using intravesical thermochemotherapy other than those applicable to cold instillations. These include urethral stricture, acute urinary infection, significant hematuria, and known drug hypersensitivity [12].

In the Czech Republic, reimbursement for Hyperthermic Intra-VEsical Chemotherapy (HIVEC) treatment is currently approved according to the criteria proposed by the Czech Urological Society; however, convincing high-quality evidence demonstrating its efficacy in treating NMIBC is lacking.

## Study population characteristic and methods

In this paper, we present the results of HIVEC treatment in a tertiary onco-urological center in a retrospective non-comparative cohort study by evaluating data from a prospectively maintained registry. Only patients with HR and very HR primary or recurrent NMIBC who were eligible for intravesical treatment were included. After initial TURB, early re-resection was performed according to European Association of Urology (EAU) guidelines or at the discretion of the operating surgeon (such as complete resection of a large Ta tumor, etc.) [3]. Immediate intravesical chemotherapy as a single one-shot treatment was not recommended in any case, especially for conditions such as large or highly recurring tumors, instances involving significant bleeding, etc. Stage and grade of the disease were assigned by an experienced uropathologists (JH) using the TNM classification approved by the Union International Contre le Cancer (UICC) (8th Edn.) and both WHO 2002/2016 and WHO 1973 grading classifications, respectively. All patients received topical adjuvant intravesical thermochemotherapy and were prospectively followed in a clinical intrainstitutional register. HIVEC was used as an alternative to intermittently unavailable BCG vaccine. Radical cystectomy was recommended to patients with the highest risk disease, but it was refused by them. The patients were recruited as they appeared consecutively one by one. If they were candidates for BCG (HR disease) or for radical cystectomy (very HR disease) but refused, they were given HIVEC. At that time, there were no other alternative options available (such as electromotive drug administration = EMDA, Synergo, Gemcitabine, TAR, etc.). Each instillation involved continuous intravesical recirculation of either Mitomycin C (MMC) 40 mg or Epirubicin 50 mg for 60 min at a temperature of 43 °C [13]. The selection of chemotherapy was based on the current availability of drugs. Since January 2020, only epirubicin has been utilized. According to the available data, the synergistic effect of a drug (chemotherapy and hyperthermia) was found to be comparable between epirubicin and MMC, both on an experimental and clinical level [14, 15]. All instillations were performed with the CombatBRSsystem V2.0 (Combat Medical, Wheathampstead, UK). The number of instillations varied based on the actual instructions and protocols provided by the manufacturer, but the minimum was 6 weekly instillations (induction), and the maximum was 15 instillations (monthly mode). Any extra instillation beyond induction was considered maintenance. The patients were followed according to EAU guidelines recommendation for HR disease, namely by cystoscopy and urinary cytology at 3 months and if negative, subsequent cystoscopy and cytology were repeated every 3 months for a period of 2 years, and every 6 months thereafter until 5 years, and then yearly. An imaging of the upper tract was done yearly [3, 16]. In case of a suspected recurrence, a TURB with tissue sampling was performed, and events [any recurrence, clinically significant recurrence (HR) = high grade (HG) and/or T1 disease and progression =  ≥T2 disease or dissemination] were recorded. Other events included radical cystectomy, death from urothelial carcinoma, and death from other causes. The primary endpoint was to evaluate the time to HR recurrence and event-free survival rates (HR recurrence or progression). Secondary endpoints were survival rates without any recurrence, the effect of maintenance on survival by comparing of the groups with and without maintenance and rate of complications. Additionally, besides the use of maintenance, survival rates without HR recurrence were analyzed taking into account other variables such as use of epirubicin/MMC, tumor size ≥3 cm, multifocality, presence of CIS, T1 stage and primary/recurrent disease. Adverse events were evaluated using the standardized methodology of Common Terminology Criteria for Adverse Events (CTCAE) version 4.0.

## Statistical analysis

Kaplan–Meier (KM) analysis was used to evaluate survival, and the curves were compared using the Logrank test. Multivariable Cox regression analysis was then used to determine the variables independently correlating with the rates of survival without a HR recurrence. Only variables significantly correlating in the Logrank test were included. The Mann–Whitney test was used to compare groups with/without maintenance therapy for age and number of instillations (continuous variables), and Fisher’s exact test was used for binomial variables. A probability value (*p*) (two sided) of <0.05 was considered statistically significant. All calculations and graphs were produced using MedCalc® Statistical Software version 20.118 (MedCalc Software Ltd, Ostend, Belgium; https://www.medcalc.org; 2022).

## Results

Between November 2014 and June 2022, a total of 47 patients were diagnosed with HR (74.5%) or very HR disease (25.5%). Eight included patients recurred after previous intravesical treatment of NMIBC: 5 patients experienced recurrence after cold intravesical chemotherapy (10.6%), and three after BCG therapy (two were BCG refractory tumors and one low-grade recurrence) (6.4%). Table 1 displays the complete study population characteristics, while Table 2 describes the characteristics of patients with and without maintenance. None of the patients experienced a recurrence in the upper tract or showed evidence of cN + on imaging during the follow-up period. Five patients underwent radical cystectomy. One woman underwent the procedure due to contractory bladder (T0), while another woman progressed to muscle-invasive disease after five instillations (final stage T1 N0 HG urothelial carcinoma). One man had a recurrence after five instillations and subsequently also after following BCG intravesical therapy. The final histology revealed T1 N0 low-grade urothelial carcinoma, with the original histology being T1 HG disease. Another man experienced a recurrence after nine instillations, with the final histology indicating T1 HG N0 in the bladder and T2 disease in the prostatic urethra. The last man had a recurrence after six instillations, reaching the final stage of T2 N0 disease with rapid dissemination into the lungs, requiring systemic oncological treatment.

**Table 1:** Study population characteristics

Number of patients (*n*)	47
Female (*n*, %); Male (*n*, %)	13 (27.7); 34 (72.3)
Age (average, min–max)	66.6 (41–87)
Primary tumor (*n*, %); Recurrent tumor (*n*, %)	17 (36.2); 30 (63.8)
Multiplicity (*n*, %), Single lesion (*n*, %)	38 (80.9); 9 (19.1)
Stage T1 (*n*, %)	30 (63.8)
Size ≥30 mm	14 (29.8)
Pathologic grade HG (*n*, %); LG (*n*, %)	35 (74.5); 12 (25.5)
Concomitant CIS (*n*, %)	9 (19.1)
Epirubicin (*n*, %); Mitomycin C (*n*, %)	10 (21.3); 37 (78.7)
Recurrence rate (*n*, %)	23 (48.9)
High-risk recurrence rate (*n*, %)	16 (34)
Rate of progression (*n*, %)	5 (10.6)
Rate of cystectomy (*n*, %)	5 (10.6)
Death from urothelial carcinoma (*n*, %)	3 (6.4)
Death from all cause (*n*, %)	10 (21.3)

**Table 2:** Groups with and without maintenance—study population characteristics

Variable	Maintenance given	Induction only	*p* value
*N* (%)	29 (61.7)	18 (38.3)	
Age—median (IQR)	69 (61.8–73.3)	71 (62–73)	0.86
Gender—*n* (%)			
Male	22 (75.9)	12 (66.7)	0.72
Female	7 (24.1)	6 (33.3)	
Risk group—*n* (%)			
High risk	19 (65.5)	16 (88.9)	0.14
Very high risk	10 (34.5)	2 (11.1)	
Number of instillations—median (IQR)	10 (9–10)	6 (6–6)	<0.0001
Type of chemotherapy—*n* (%)			
Mitomycin C	20 (69)	17 (94.4)	0.08
Epirubicin	9 (31)	1 (5.6)	
Allergy—*n* (%)			
Yes	2 (6.9)	0	0.76
No	27 (93.1)	18 (100)	

The median time of follow-up without an event was 32 months (IQR: 8.5–57). Of those experiencing an event, the median time to any recurrence was 9 months (IQR: 7.3–16.8), while the median time to HR recurrence and progression were 15 months (IQR: 8–24) and 11 months (IQR: 4.5–25.3), respectively.

**Fig. 1:**
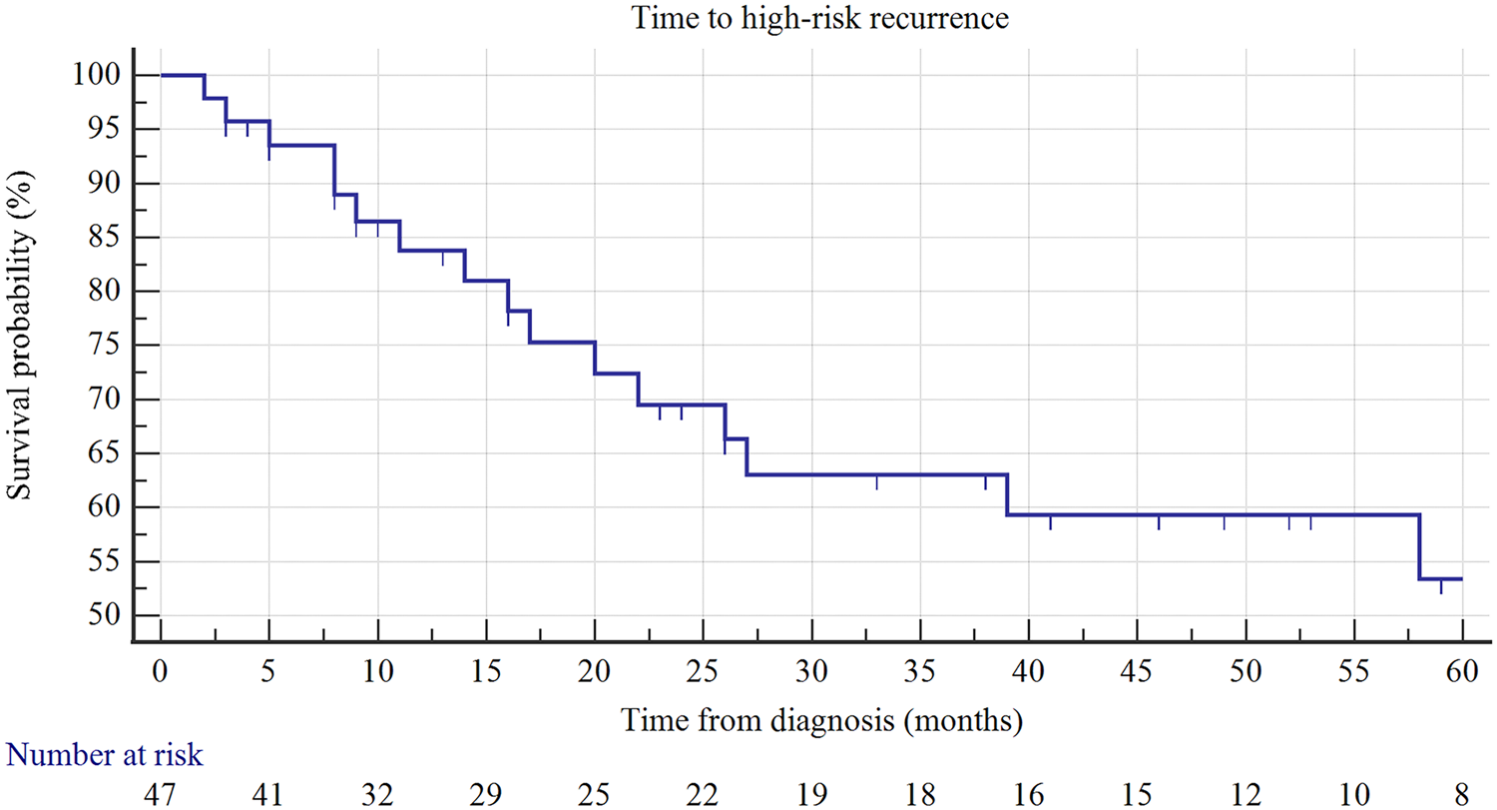
Kaplan–Maier curve of HR RFS

**Fig. 2:**
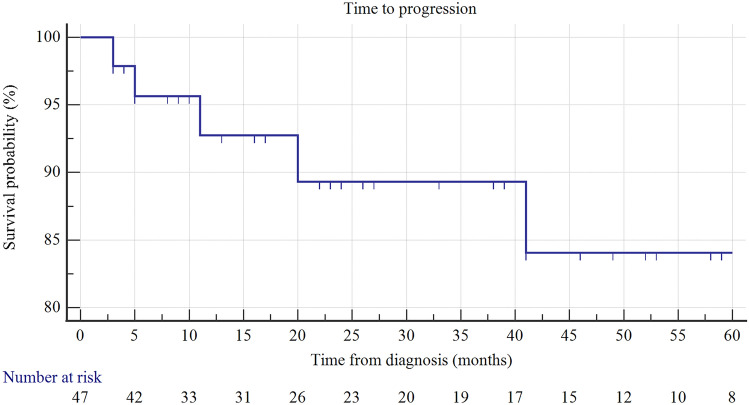
Kaplan–Maier curve of PFS

The HR RFS (recurrence-free survival) rates at 12, 24, and 48 months was 84, 70, and 59%, respectively (KM analysis) (Fig. 1). After two years of follow-up, 89% of patients survived without progression (PFS rates) (Fig. 2), as well as without cystectomy.

**Fig. 3:**
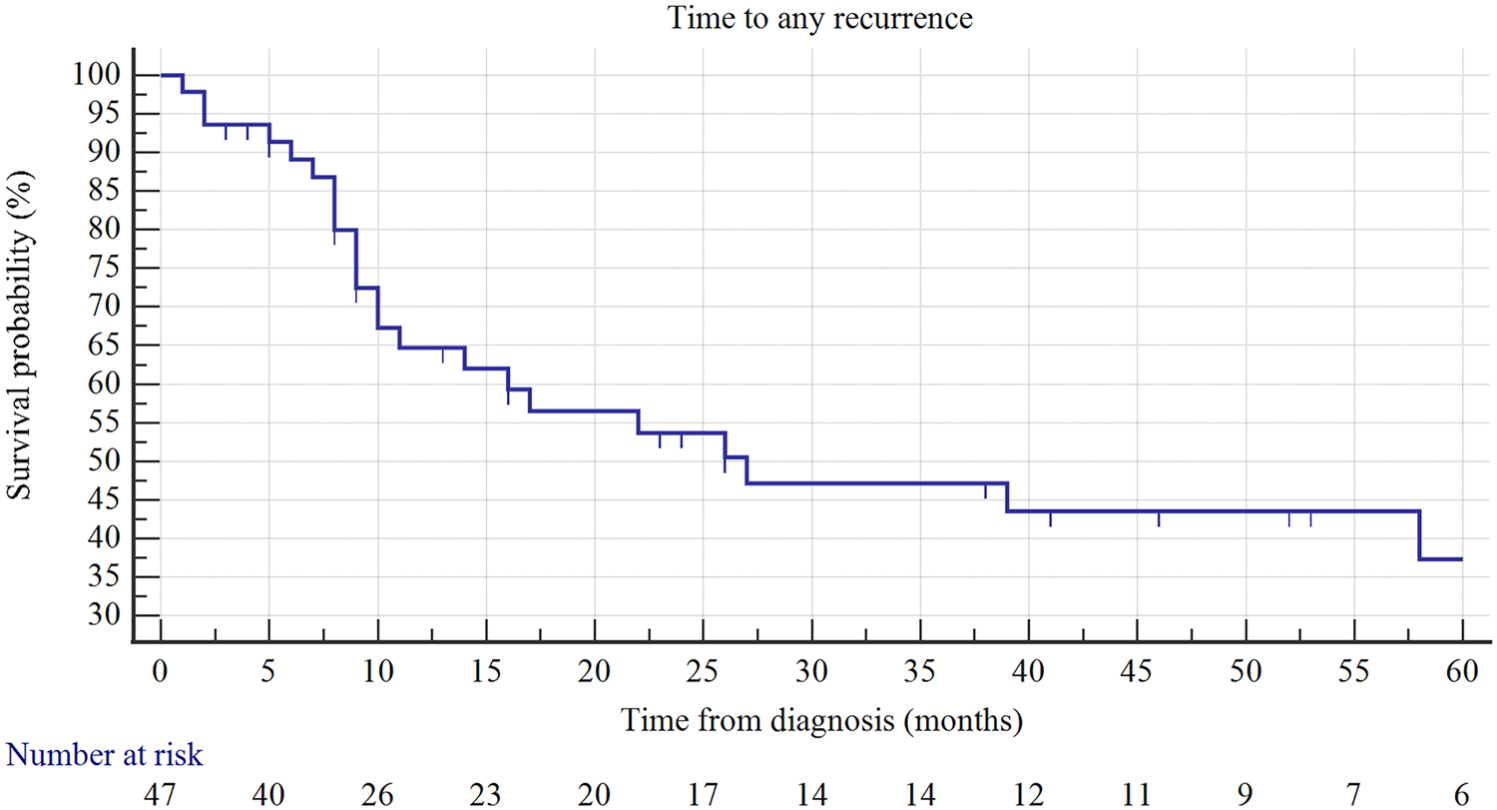
Kaplan–Maier curve of RFS

**Fig. 4:**
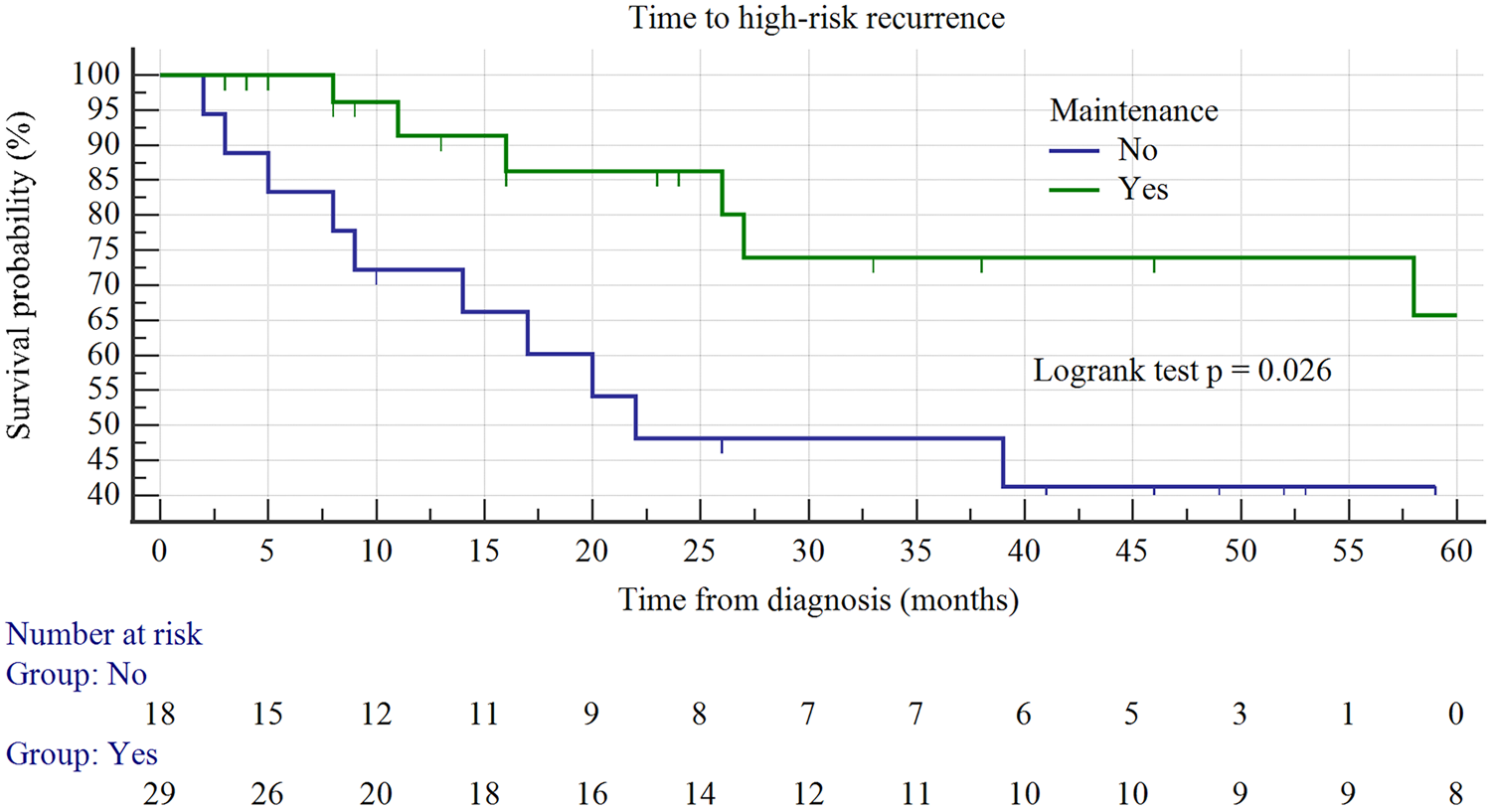
Kaplan–Maier curves of HR RFS with/without a maintenance

The RFS rates for any recurrence was 65, 54, and 44% at 12, 24, and 48 months, respectively (Fig. 3). Maintenance therapy was a significant factor in improving the survival rate without a HR recurrence (Fig. 4). Two years after receiving maintenance therapy, 86% of patients survived without HR recurrence, while only 48% of patients who only received induction survived (*p* = 0.026). Moreover, taking into account other clinical and histopathological variables (use of epirubicin/MMC, tumor size ≥3 cm, multifocality, presence of CIS, T1 stage and primary/recurrent disease), only presence of CIS adversely correlated with survival without a HR disease in the Logrank test (*p* = 0.01). In a multivariable Cox regression analysis, both the use of maintenance and the presence of CIS independently correlated with survival without a HR recurrence (Table 3).

**Table 3:** Multivariate Cox regression analysis demonstrating correlations with risk of HR recurrence

	Hazard ratio	95% CI of hazard ratio	*p* value
Maintenance given	0.34	0.12–0.96	0.042
CIS present	3.12	1.16–8.41	0.025

A total of 9 patients (19.1%) were subsequently given BCG treatment due to recurrence, while 5 patients underwent radical cystectomy (10.6%).

Similarly 9 patients (19.1%) experienced low-grade G1-2 complications that were transient and did not lead to treatment termination or its longer interruption. One patient with a history of radiotherapy of the cervix developed a contractile bladder later after the thermoinstillation, which finally required radical cystectomy. A toxoallergic skin reaction to MMC was recorded in 2 patients, while another patient developed it early after starting treatment and, thus, was not included in the evaluation. An overview of side effects is presented in Table 4.

**Table 4:** Overview of the adverse events classified according to Common Terminology Criteria for Adverse Events (CTCAE) version 4.0

Study therapy related AEs	Grade 1–2	Grade 3	Grade 4–5
LUT infection	2 (4.3%)	–	–
Allergy	3 (6.3%)	–	–
Bladder spasm/cramp	1 (2.1%)	–	–
Urgency	7 (14.9%)	–	–
Bladder pain	1 (2.1%)	–	–
Contractile bladder	–	1 (2.1%)	–

## Discussion

The first randomized prospective studies were designed for intermediate-risk tumors treated with HIVEC as a potentially more effective method than the recommended adjuvant “cold” intravesical chemotherapy [17]. The HIVEC II study did not demonstrate the benefit of thermotherapy in the form of 6 weekly thermoinstillation with 40 mg MMC compared to six classical treatments, i.e., without maintenance schedules [18]. RFS rate after 24 months of follow-up was comparable in both arms, and the probability of completing treatment was significantly lower in the arm with thermotherapy (59 vs. 89%). Lower urinary tract complications of grade 1–2 were recorded in 66% of patients in the intervention group and 69% of patients in the control group. Serious complications were rare in both groups. Therefore, attention was focused on treating patients at high risk, especially in the context of the situation created by the limited availability of BCG vaccine, as the standard and most effective treatment for this indication so far. Generally, there is either no or limited high-quality data on the use of HIVEC in these patient populations, including primary or concomitant CIS. Data from prospectively conducted registries involving 14 European centers demonstrated results similar to BCG data based on EORTC nomograms. Specifically, RFS rates and PFS rates were reported at 82 and 98% of patients after an average follow-up time of 21 months [19, 20]. In comparison to this extensive patient dataset, our results were less favorable, with a 24-month RFS rates and PFS rates of 54 and 89%, respectively. This discrepancy can be attributed to the high involvement of very HR disease patients (25.5%) in our group, including those with recurrences after BCG therapy, cold intravesical chemotherapy, and with CIS. In the only pilot prospective randomized phase 2 study, a total of 50 patients with HR NMIBC without CIS were evenly divided into two arms of 25 patients [21]. In one arm, classic BCG vaccine was administered, including maintenance therapy for one year, while the other arm received thermochemotherapy with MMC 40 mg using the Combat BRS system for a total treatment duration of 6 months, including maintenance doses. After 24 months, 95% of patients treated with HIVEC were disease-free compared to 75.1% of patients treated with BCG (*p* = 0.064) in a per-protocol analysis (statistically insignificant result). Progression was zero in the HIVEC arm after 2 years, while 24.9% in the BCG arm (*p* = 0.018, per-protocol analysis). Our study results exhibit trends similar to the BCG subgroup, with a 24-month RFS rates of 54%, HR RFS rates of 70%, and PFS rates of 89%, respectively. It is important to note that 38% of the patients in our group received only induction treatment without maintenance. Additionally, we included patients with CIS as a prognostic factor, which has been shown to be associated with a poorer response in patients with NMIBC failing after BCG [15]. Both variables (use of maintenance and presence of CIS) adversely correlated with survival without a HR recurrence in our study. Concerning the adverse events in the study by Guerrero-Ramos, Grade 1 complications occurred in 33.3% of HIVEC cases and 20.8% of BCG cases, while grade 3 complications were comparable (16.7% in both groups). In another retrospective single-arm study, the authors reported adverse events in 35.1% of patients. The most frequently documented events were rash (10.5%), urinary tract infection (8.8%), and bladder spasm (8.8%) [22]. In comparison, only 19.1% of our patients experienced G1-2 complications, and 2.1% experienced G3 complications. There were no discernible trends indicating an increased frequency or urgency of G2-3 complications associated with the use of epirubicin, as observed by other authors [15]. Guerrero-Ramos et al. concluded that HIVEC with maintenance therapy provides comparable results to BCG and is a reasonable alternative during periods of BCG shortage. However, a significant limitation remains the very low number of patients in both arms and the short follow-up period.

When compared to data from current large patient cohorts with HR tumors after BCG therapy, the numbers are similar. For example, Matulay reported data on 542 patients with NMIBC treated with BCG, of which 90% had HR tumors (the remainder had intermediate-risk tumors) and 32% had CIS on histology [6]. With a median follow-up of 47.8 months, HG RFS rates at 1, 3, and 5 years were 81, 76, and 74%, respectively. PFS rate was 93% after 3 years of follow-up. In our study, the HR RFS rates at 12, 24, and 48 months was 84, 70, and 59%, respectively, and the PFS rates was 89% after 2 years. It should be noted that our cohort had half the follow-up time and a significantly lower number of patients, but all had HR tumors, with 25.5% having very HR tumors, including recurrence after BCG and intravesical chemotherapy and CIS.

The benefit of maintenance beyond six instillations has been demonstrated in our study, highlighting its positive effect on prognosis similar to BCG maintenance treatment [23]. However, there are limited data on thermochemotherapy. In a study by Plata et al., a duration of adjuvant HIVEC therapy of less than four months was associated with an increased risk of recurrence (HR 1.72) [24].

It is noteworthy that during the follow-up of patients after thermochemotherapy, cytopathological changes in urine can be observed, mimicking cancer cells. In a study by Pierconti, urine cytology was investigated in patients undergoing EMDA and HIVEC treatment for BCG-unresponsive disease. An increase in cellularity and nuclear size with alterations in the nuclear/cytoplasmic ratio was common in patients treated with EMDA/MMC and HIVEC/MMC, even without clinical and histological evidence of neoplasia recurrence. Hyperchromasia and irregular nuclear chromatin were rarely observed, and the irregular nuclear membrane was a feature present in urine cytology after C-HT/MMC treatment [25].

Limitations of the study, in addition to those mentioned above, include its single-arm design and retrospective data evaluation. However, these limitations were reduced by a prospectively managed register including reporting of adverse events. Furthermore, the patient cohort was heterogenous, and an adequate number of instillations for each patient group was not defined at the time. The risk of selection bias is also not negligible, even though the patients were included consecutively, with HIVEC being the only alternative to BCG and radical cystectomy.

## Conclusion

In conclusion, based on our experience, HIVEC including a maintenance can be considered a safe and effective alternative to BCG during times of its limited availability. Our results cannot be interpreted as a recommendation for routine use of HIVEC in the treatment of NMIBC until high-quality data is available. However, the presented data may serve as a basis for a prospective, randomized study comparing BCG and HIVEC in these patient populations.

## Data Availability

The data that support the findings of this study are available on request from the corresponding author, [AB].

## Declarations

### Major statement

The authors present an original article demonstrating the results of intravesical thermochemotherapy used in patients with high and very high-risk non-muscle-invasive urothelial bladder cancer as an alternative to intravesical Bacillus Calmette–Guérin (BCG) vaccine and radical cystectomy.
